# Development and internal validation of a risk model for hyperuricemia among people living with HIV in Hangzhou, China: a retrospective longitudinal cohort study

**DOI:** 10.3389/fendo.2026.1736775

**Published:** 2026-04-16

**Authors:** Wenhui Zhang, Jinchuan Shi, Shourong Liu, Er Li, Yan Zhang, Ying Wang, Dingyan Yan, Jianhua Yu, Yi Wang

**Affiliations:** 1 Department of Infection, Affiliated Hangzhou Xixi Hospital, Zhejiang Chinese Medical University, Hangzhou, China; 2 Department of Nursing, Affiliated Hangzhou Xixi Hospital, Zhejiang Chinese Medical University, Hangzhou, China; 3 Medical Laboratory, Affiliated Hangzhou Xixi Hospital, Zhejiang Chinese Medical University, Hangzhou, China; 4 Clinical Research Laboratory, Affiliated Hangzhou Xixi Hospital, Zhejiang Chinese Medical University, Hangzhou, China

**Keywords:** hyperuricemia, nomogram, people living with HIV, prediction model, risk factor

## Abstract

**Background:**

The aim of this study was to identify independent risk factors for the time to hyperuricemia (HUA) in PLWH and develop an HUA risk model based on a retrospective study in Hangzhou, China.

**Methods:**

PLWH treated at Hangzhou Xixi Hospital between July 2016 and December 2024 were randomly assigned to training and validation cohorts in a 7:3 ratio. Independent risk factors associated with the occurrence of HUA were identified using multivariable Cox regression analysis. Characteristic variables for constructing the HUA risk prediction model were selected through the least absolute shrinkage and selection operator (LASSO) method, and the model was visualized using a nomogram. The model’s performance was assessed through time-independent receiver operating characteristic (ROC) curve, calibration curve, concordance index (C-index), and decision curve analysis (DCA) to evaluate its discriminatory ability, calibration, and clinical applicability. A generalized linear mixed model (GLMM) was used to investigate the effects of different ART regimens on the SUA levels over time among PLWH.

**Results:**

A total of 631 participants were included in this study, with 439 assigned to the training set and 192 to the validation set. The final model for predicting HUA risk in PLWH incorporated two factors: baseline serum uric acid (SUA) levels and baseline CD4^+^ T-cell count. The area under the ROC curve (AUC) values for 1-, 3-, and 5-year HUA risks were 0.72, 0.74, and 0.75 in the training set, and 0.66, 0.67, and 0.70 in the internal validation sample, respectively. The calibration curve demonstrated strong agreement between predicted and observed outcomes. Both the C-index and DCA confirmed the nomogram’s superior predictive performance. Furthermore, the prevalence of hypercholesterolemia and abnormal eGFR differed significantly between the defined high- and low-risk groups. Comparing with PLWH receiving the B/F/TAF regimens, there was a significant decrease in SUA values over time in PLWH receiving EFV-containing ART regimens.

**Conclusions:**

We established and validated a HUA-specific nomogram for predicting the risk of HUA in PLWH. This model provides clinicians with a practical tool for the early-stage identification of PLWH at high risk of HUA, a capability that is highly significant for guiding clinical treatment.

## 1 Introduction

Hyperuricemia (HUA), characterized by elevated serum uric acid (SUA) levels, has been associated with cardiovascular diseases (CVD), a leading cause of morbidity and mortality among people living with HIV (PLWH) (1, 2). In addition to CVD, this condition is also linked to the presence and severity of chronic diseases, including gout (3), metabolic syndrome (4), CVD (5), diabetes mellitus (6), and kidney disease (7), thereby imposing a substantial public health burden (8). Multiple risk factors contribute to HUA, such as a high-purine diet, medication use, hypertension, hypothyroidism, and obesity. Social determinants, including higher socioeconomic status and a history of smoking and alcohol use, further increase the risk of developing this condition (9–12).

First described in 1993, HUA is among the most common rheumatic manifestations of HIV infection (11). Despite this early recognition, research aimed at understanding HUA in this vulnerable population has seen little progress over the subsequent decades. This limited research focus is particularly critical in sub-Saharan Africa, where studies addressing the burden of HUA among PLWH have only recently begun to emerge. The prevalence rates of HUA in PLWH reported in previous literature vary considerably, ranging from 13% to 46.5%. This significant heterogeneity underscores the urgent need for robust research in this domain (1).

Recent studies have established a link between specific antiretroviral therapy (ART) regimens and HUA in PLWH. The ART agents implicated in HUA include the nucleoside reverse transcriptase inhibitors tenofovir disoproxil fumarate (TDF) and lamivudine (3TC); the non-nucleoside reverse transcriptase inhibitor efavirenz (EFV); the integrase inhibitor dolutegravir (DTG); and the protease inhibitor ritonavir-boosted atazanavir (ATV/r) (13, 14). Despite this, few studies have addressed the prevalence of HUA and its associated risk factors in PLWH following ART initiation. Kwesiga et al. (11) determined that current alcohol consumption and an elevated respiratory rate were independently associated with HUA. Gout is the major manifestation of HUA, and, in 2012, one study identified several factors associated with its occurrence among PLWH, namely, male sex, black African ethnicity, high body mass index (BMI), hypertension diagnosis and treatment, and treatment for hypertriglyceridemia (13). However, to date, no risk prediction model for the time to HUA has been developed and validated within the current clinical context of widespread use of diverse ART regimens among PLWH.

HUA warrants close attention as a critical intermediary event in the progression toward adverse health outcomes, as it not only regulates metabolic alterations *in vivo* but also independently precipitates cardiovascular events. Thus, the primary objective of this study was to establish an effective prediction model for HUA in PLWH through regression analysis of a large-scale cohort. This model is expected to provide a theoretical foundation for clinical decision-making and health intervention strategies, ultimately aiming to reduce adverse prognostic outcomes among PLWH.

## 2 Materials and methods

### 2.1 Study population and design

This retrospective study included a cohort of 1034 PLWH admitted to Hangzhou Xixi Hospital. All participants received ART-initiated therapy and follow-up between July 1, 2016 and December 23, 2024, at Hangzhou Xixi Hospital, the largest AIDS designated hospital in Zhejiang Province, China.

The inclusion criteria were as follows: (1) confirmed HIV infection verified by western blot; (2) age ≥ 18 years; (3) receipt of ART; (4) followed up between 2016 and 2025; (5) initial treatment regimen consisting of TDF/zidovudine (AZT) + 3TC + EFV, TDF + 3TC + DTG, TDF + 3TC + nevirapine (NVP), or bictegravir/emtricitabine/tenofovir alafenamide (B/F/TAF); (6) availability of baseline data including SUA, triglycerides, cholesterol, glomerular filtration rate (GFR), blood glucose, high-density lipoprotein cholesterol (HDL-C), and low-density lipoprotein cholesterol (LDL-C). The exclusion criteria included: (1) a history of cancer; (2) systemic infection; (3) pregnancy; (4) unknown initial ART regimen; (5) HUA at baseline; (6) incomplete clinical data.

### 2.2 Data collection

**Figure 1:**
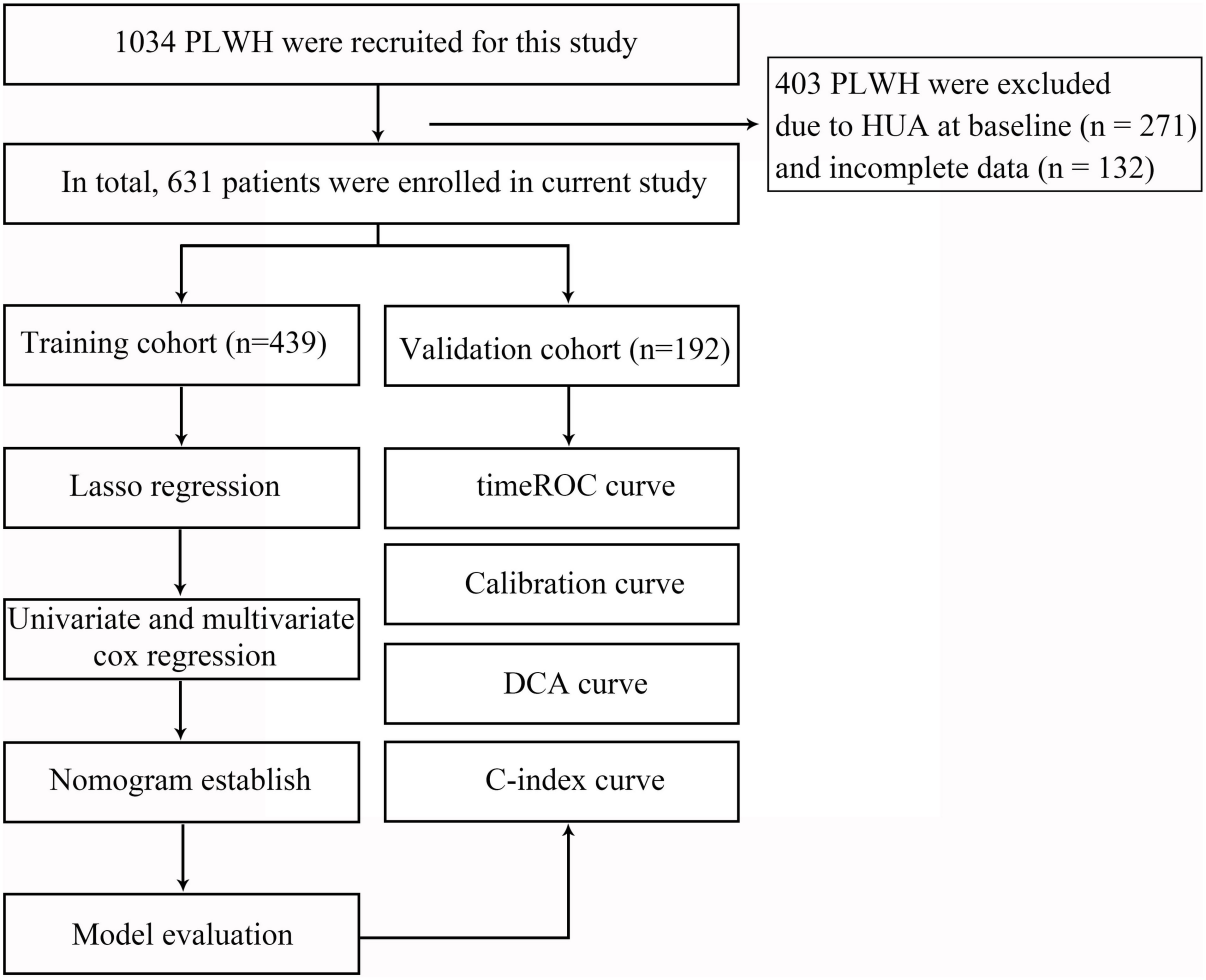
Flowchart of all participants in the study. LASSO, the least absolute shrinkage and selection operator; timeROC, time-independent receiver operating characteristic; DCA, decision curve analysis; C-index, concordance index.

Baseline data were obtained from the Electronic Medical Records (EMR) management system and included demographic characteristics (age, sex, marital status, education level, BMI, HIV transmission route, smoking status, and drinking status), as well as disease-related indicators such as baseline HIV RNA levels, baseline CD4^+^ T cell counts, and WHO clinical stage. The study flowchart is presented in **Figure 1**. The SUA levels were measured at baseline and at 1, 2, 3, and 4 years after ART initiation.

### 2.3 Definition of metabolic disorders

Hypertriglyceridemia (HTG) was defined as TG levels ≥ 2.26 mmol/L (15). High total cholesterol (HTC) was defined as total cholesterol (TC) ≥ 6.22 mmol/L (16). Elevated low-density lipoprotein cholesterol (high LDL-C) was defined as LDL-C ≥ 4.14 mmol/L (17). Low high-density lipoprotein cholesterol (low HDL-C) was defined as HDL-C ≤ 1.03 mmol/L in men and ≤ 1.29 mmol/L in women (11). Hypertension was defined as systolic blood pressure (SBP) ≥ 140 mmHg and/or diastolic blood pressure (DBP) ≥ 90 mmHg, or current use of antihypertensive medication (11). Elevated fasting plasma glucose (FPG), indicating dysglycemia, was defined as FPG ≥ 5.6 mmol/L (18). An estimated glomerular filtration rate (eGFR) < 90 mL/(min·1.73 m^2^) was considered indicative of impaired renal function (19).

### 2.4 Definition of hyperuricemia

The definition of HUA was based on the concentration of SUA. The diagnostic criterion for HUA was an SUA level exceeding 420 μmol/L (3, 20).

### 2.5 Outcomes of interest

The primary outcome of this study was the identification of risk factors for HUA. The secondary outcome was the comparison of dynamic changes in SUA levels across the three ART regimens (DTG-containing, EFV-containing, and B/F/TAF regimens).

### 2.6 Predictor selection

Least absolute shrinkage and selection operator (LASSO) Cox regression was applied for data dimensionality reduction and the identification of optimal predictors for constructing the nomogram (21). Analysis was conducted using the “glmnet” package in R software, with the model parameter set to “family = Cox” (22).

### 2.7 Construction of model

A nomogram was generated to visualize the predictive model, which was derived from multivariate Cox regression analysis performed using the “rms” package in R. Each predictor was assigned a score based on its standardized regression coefficient, forming a scoring system for all variables (21). The total score, calculated by summing individual predictor scores, was used to estimate the probability of HUA for each PLWH (1-year, 3-year, 5-year). A higher total score was associated with a worse overall survival (OS).

### 2.8 Performance and validation of model

For internal validation, we used a train-test split based on individual persons. In each dataset, a random sample of 70% of persons was used to develop the prediction models and the remaining 30% were used to internally validate the models.

The predictive performance of the risk model was evaluated using time-dependent receiver operating characteristic (ROC) curve analysis in training data set. The ROC curves for predicting 1-, 3-, and 5-year overall survival were generated using the “timeROC” package in R (23). The greater the AUC value, the larger the area under the curve, and the more accurate the forecast. Calibration curves were constructed using the “rms” package to assess the agreement between predicted and observed outcomes in training data set, thereby reflecting the model’s calibration capability (24). The C-index and DCA were further applied to quantify the discriminative ability and clinical utility of the model in training data set, respectively, by estimating the net benefit across a range of threshold probabilities (25). In addition, we examined the internal validation performance using ROC, calibration curves, C-index and DCA on the entire internal validation data set.

### 2.9 Statistical analysis

All statistical analyses were performed using R statistical software (v4.1.3) and IBM SPSS Statistics (v25.0). Normally distributed continuous variables are presented as means with standard deviations. Non-normal variables are described as medians with interquartile ranges. Categorical variables are reported as numbers and percentages, while proportions were compared using Pearson’s chi-squared test or Fisher’s exact test. All line graphs were generated using GraphPad Prism version 8.0.2 (263) (GraphPad Software, San Diego, CA, USA).

IBM SPSS Statistics (v25.0) was used to randomly divide the research data into a training set and a validation set in a 7:3 ratio. A hazard ratio (HR) greater than or less than 1 was interpreted as an increased or decreased association with HUA, respectively. The “Survival” and “Survminer” packages in R were used to analyze HUA-related data among PLWH. Kaplan-Meier survival curves were drawn to compare PLWH with and without HUA, and differences were compared using the log-rank test. A generalized linear mixed model (GLMM) was used to investigate the effects of different ART regimens on the SUA levels over time among PLWH. A *P*-value of < 0.05 was considered statistically significant.

## 3 Results

### 3.1 Baseline characteristics of the study population

Based on the inclusion and exclusion criteria, a total of 1,034 PLWH were initially screened, of whom 403 were excluded due to HUA at baseline (n = 271) and incomplete clinical data (n = 132). Ultimately, 631 PLWH were included in the longitudinal cohort analysis (**Figure 1**). The mean age of the participants was 35.0 years (SD: 12.8), and 91% were male. The mean BMI was 22.3 kg/m^2^ (SD: 3.0). The mean baseline CD4^+^ T-cell count was 333.6 cells/μL (SD: 193.4). The ART regimens consisted of EFV-based therapy (AZT/TDF + 3TC) for 311 participants, DTG-based therapy (AZT/TDF + 3TC or 3TC) for 110 participants, B/F/TAF for 158 participants, and other regimens for 52 participants. After follow-up, the overall prevalence of HUA was 41.83% (264/631). By switching status, the prevalence was 58.25% (226/388) among PLWH who switched ART regimens (n = 388) and 15.64% (38/243) among those who did not (n = 243). Additional demographic characteristics, comorbidities, and laboratory indices are summarized in **Table 1**.

**Table 1:** Baseline characteristics of all participants (n = 631).

Characteristics	Values
Age at initial treatment (years)	35.0 ± 12.8
Gender, n (%)	
Male	574 (91.0)
Female	57 (9.0)
BMI (Body mass index)	22.3 ± 3.0
Education level, n (%)	
High school or below	237 (37.6)
College/Associate degree	146 (23.1)
Bachelor’s degree or higher	248 (39.3)
Marital status, n (%)	
Married	135 (21.4)
Divorced or widowed	77 (12.2)
Unmarried	419 (66.4)
Transmission route, n (%)	
Heterosexual	222 (35.2)
Homosexual	398 (63.1)
Other	11 (1.7)
Smoking history, n (%)	
Never	392 (62.1)
Occasional	91 (14.4)
Daily	148 (23.5)
Alcohol consumption, n (%)	
Never	348 (55.2)
Occasional	264 (41.8)
Daily	19 (3.0)
Hypertension, n (%)	
No	534 (84.6)
Yes	97 (15.4)
Baseline CD4 count (cells/μL)	333.6 ± 193.4
Baseline HIV-RNA (copies/mL)	
< 500,000	120 (19.0)
≥ 500,000	431 (68.3)
missing	80 (12.7)
Baseline LDL-C	2.3 ± 0.6
Baseline TG	1.3 ± 0.8
Baseline HDL-C	1.0 ± 0.2
Baseline SUA	343.5 ± 54.3
Baseline GLU	5.5 ± 1.1
Baseline TC	4.1 ± 0.8
Baseline eGFR	115.8 ± 20.2
Initial ART regimen, n (%)	
EFV (AZT/TDF+3TC)	311 (49.3)
DTG (AZT/TDF+3TC or 3TC)	110 (17.4)
B/F/TAF	158 (25.0)
Others (TDF + 3TC+ANV/LPV/r, EVG/c/FTC/TAF, AZT/3TC+LPV/r)	52 (8.3)
Prevalence of HUA (after follow-up), n (%)	
Total (n =631)	264 (41.83)
ART-switching group (n = 388)	226 (58.25)
Non-switching group (n = 243)	38 (15.64)

BMI, body mass index; LDL-C, low-density lipoprotein cholesterol; TG, triglycerides; HDL-C, high-density lipoprotein cholesterol; TC, total cholesterol; SUA, serum uric acid; GLU, glucose; eGFR, estimated glomerular filtration rate; HUA, hyperuricemia; ART, antiretroviral therapy.

### 3.2 Variable selection and risk model construction

The 631 eligible participants were randomly allocated into training (*n* = 439, 70%) and validation (*n* = 192, 30%) sets (**Figure 1**). The baseline characteristics of the two cohorts showed no statistically significant differences (all *P* > 0.05), confirming that randomization was balanced. The demographic and clinical features are detailed in **Table 2**.

**Table 2:** Demographic characteristics of PLWH in training and validation cohorts.

Characteristics	Training set (N = 439)	Validation set (N = 192)	*P* value
Age at initial treatment (years)			
≤ 50	362 (82.46)	161 (83.85)	0.669
> 50	77 (17.54)	31 (16.15)	
Gender, n (%)			
Male	400 (91.12)	174 (90.63)	0.843
Female	39 (8.88)	18 (9.38)	
Education level, n (%)			
High school or below	163 (37.13)	74 (38.54)	0.906
College/Associate degree	101 (23.01)	45 (23.44)	
Bachelor’s degree or higher	175 (39.86)	73 (38.02)	
Marital status, n (%)			
Married	103 (23.46)	32 (16.67)	0.912
Divorced or widowed	50 (11.39)	27 (14.06)	
Unmarried	286 (65.15)	133 (69.27)	
Transmission route, n (%)			
Heterosexual	160 (36.45)	62 (32.29)	0.570
Homosexual	272 (61.96)	126 (65.63)	
Other	7 (1.59)	4 (2.08)	
Smoking history, n (%)			
Never	272 (61.96)	120 (62.5)	0.912
Occasional	65 (14.81)	26 (13.54)	
Daily	102 (23.23)	46 (23.96)	
Alcohol consumption, n (%)			
Never	241 (54.9)	107 (55.73)	0.785
Occasional	186 (42.37)	78 (40.63)	
Daily	12 (2.73)	7 (3.65)	
Hypertension, n (%)			
No	367 (83.60)	167 (86.98)	0.279
Yes	72 (16.40)	25 (13.02)	
BMI	22.24 ± 3.05	22.30 ± 2.90	0.828
Baseline CD4 count (cells/μL)	327.0 (203.0, 455.0)	310.5 (176.3, 437.8)	0.164
Baseline HIV-RNA (copies/mL)			
< 500,000	88 (20.05)	32 (16.67)	0.169
≥ 500,000	290 (66.06)	141 (73.44)	
missing	61 (13.90)	19 (9.9)	
Baseline SUA	353.1 (311.0, 387.5)	342.3 (302.6, 389.4)	0.330
Baseline GLU	5.3 (4.9, 5.7)	5.3 (4.9, 5.7)	0.190
Baseline eGFR	114.0 (102.0, 128.0)	114.0 (102.0, 130.8)	0.730
Baseline TG	1.1(0.9, 1.5)	1.1(0.8, 1.6)	0.938
Baseline TC	4.1(3.5, 4.7)	4.0 (3.5, 4.6)	0.519
Baseline HDL-C	1.0(0.9, 1.2)	1.0(0.9, 1.1)	0.638
Baseline LDL-C	2.3(1.9, 2.7)	2.3(1.9, 2.6)	0.261

BMI, body mass index; TG, triglyceride; TC, total cholesterol; LDL-C, low-density lipoprotein cholesterol; HDL-C, high-density lipoprotein cholesterol; GLU, glucose; eGFR, estimated glomerular filtration rate.

**Figure 2:**
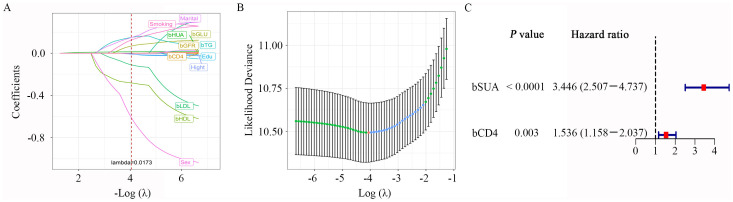
LASSO and multivariate Cox regression analysis. **(A)** Plot of partial likelihood deviance; **(B)** plot of LASSO coefficient profiles. Each color curve represents the LASSO coefficient profile of a feature against the Log (λ) sequence. The values above the figure represent the numbers of variables included in the model, with the corresponding λ shown on the x-axis. λ, lambda. **(C)** Forrest plot of multivariate Cox regression analysis. bSUA, baseline serum uric acid; bCD4, baseline CD4+ T-cell count.

Variables with a *P*-value of less than 0.1 in the LASSO regression analysis were incorporated into the subsequent univariate and multivariable Cox regression. As shown in **Figure 2**, variable coefficients consistently decreased as the lambda (λ) value increased. The optimal regularization parameter λ was selected through cross-validation, leading to the choice of λ_min_ = 0.0173 (**Figures 2A, B**). Finally, six optimal parameter values were identified and retained the further analysis.

We first calculated the cut-off values for three continuous variables that were significantly associated with the outcome of HUA. The optimal cut-off value for the SUA level was > 349.1 μmol/L, with a sensitivity and specificity of 72.96% and 62.14%, respectively. The optimal cut-off value for the baseline CD4^+^ T-cell count was ≤ 270 cells/μL, showing 45.41% sensitivity and 65.02% specificity. Height had an optimal cut-off value of > 166 cm, with a sensitivity and specificity of 86.73% and 22.22%, respectively (**Supplementary Table 1**).

Univariate and multivariable Cox regression analysis identified two significant variables: baseline SUA levels and baseline CD4^+^ T-cell counts (both *P* < 0.05, **Supplementary Table 2**; **Table 3**). As illustrated in the forest plots in **Figure 2C**, elevated baseline SUA levels (HR = 3.446; 95% CI: 2.507–4.737; *P* < 0.0001) and lower baseline CD4^+^ T-cell counts (HR = 1.536; 95% CI: 1.158–2.037; *P* = 0.003) were significantly associated with an increased risk of HUA.

**Table 3:** Multivariate Cox proportional hazards models for factors predicting the time to HUA in PLWH.

Variables	HR (95% CI)	*P* value
Height (> 166 cm)	1.199 (0.788–1.824)	0.396
Baseline SUA (> 349.1 μmol/L)	3.438 (2.501–4.726)	< 0.0001
Baseline CD4 (≤ 270 cells/μL)	1.533 (1.156–2.033)	0.003

Cm, centimeter; HR, hazard ratio; CI, confidence interval; CD4, CD4+ T cells; SUA, serum uric acid.

Subsequently, ROC curve analysis was performed to assess the discriminative performance of predictive models comprising these variables. As summarized in **Table 4**, the combined model (Model 1), incorporating both baseline SUA levels and the CD4^+^ T-cell count, demonstrated the highest predictive accuracy for HUA, with areas under the ROC curve (AUCs) of 0.72 (95% CI: 0.67–0.77), 0.74 (95% CI: 0.68–0.81), and 0.75 (95% CI: 0.64–0.86) for 1-, 3-, and 5-year predictions, respectively. In comparison, the AUC values for Model 2 (SUA only) and Model 3 (CD4^+^ T cells only) were consistently lower across all time points.

**Table 4:** Predictive performance of different models for HUA onset at 1, 3, 5 years.

Model	Variable	1-year AUC (95% CI)	3-year AUC (95% CI)	5-year AUC (95% CI)
Model1	Baseline SUA+ CD4	0.72 (0.67–0.77)	0.74 (0.68–0.81)	0.75 (0.64–0.86)
Model2	Baseline SUA	0.69 (0.64–0.74)	0.71 (0.64–0.78)	0.74 (0.65–0.84)
Model3	Baseline CD4	0.55 (0.50–0.60)	0.55 (0.49–0.62)	0.52 (0.41–0.62)

AUC, area under the curve; CI, confidence interval; CD4, CD4+ T cells; SUA, serum uric acid.

### 3.3 Performance and evaluation of risk model

**Figure 3:**
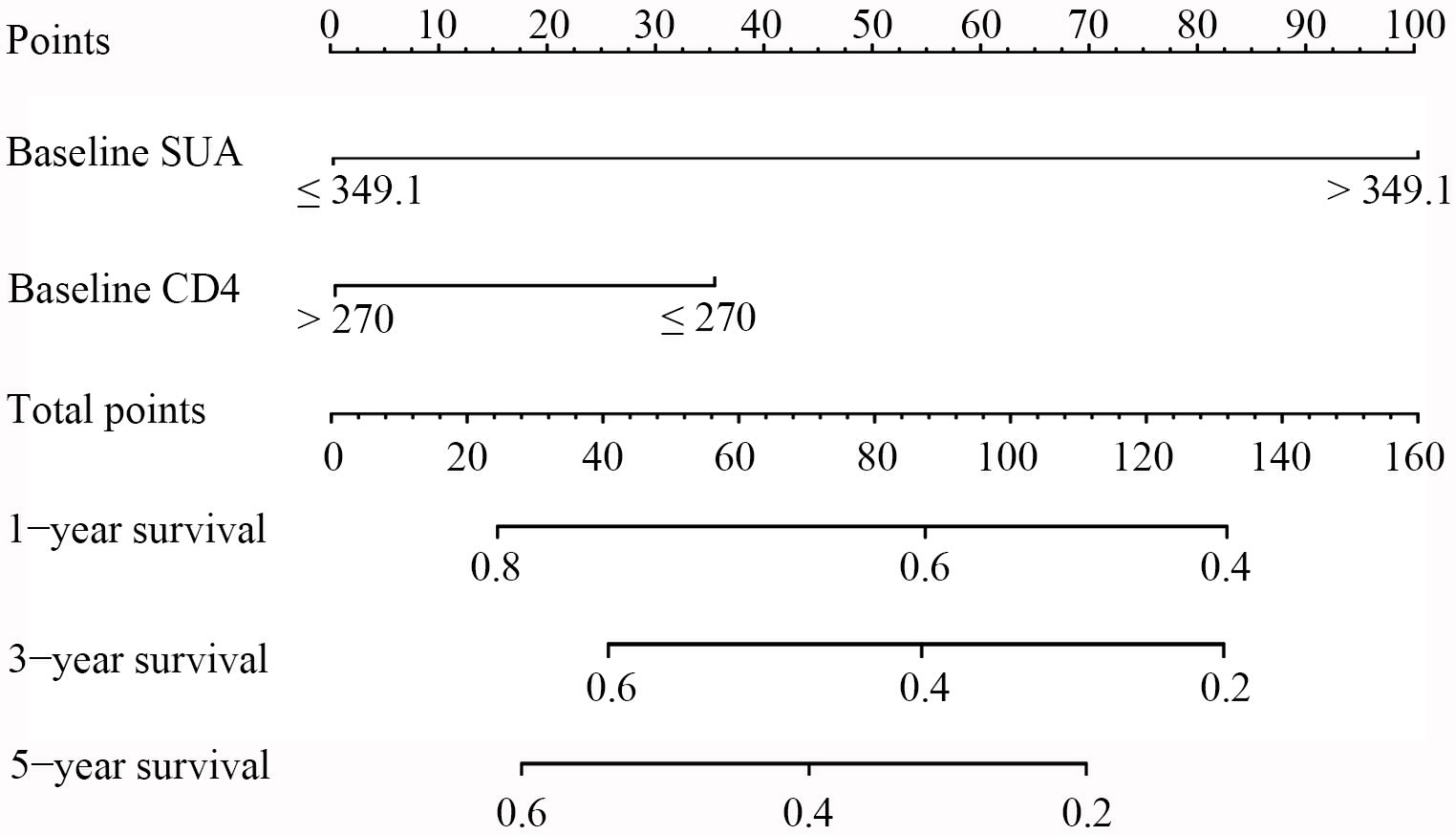
Nomogram for evaluation of HUA in PLWH. To use the nomogram, a line was first drawn from each parameter value to the score axis for the score; then the points for all the parameters were added. Finally, a line from the total score axis was drawn to determine the occurrence of HUA on the lower line of the nomogram. SUA, serum uric acid; CD4, CD4+ T-cell count.

Based on the above findings, two variables including baseline CD4^+^ T-cell counts and baseline SUA level were incorporated into a risk prediction nomogram to estimate the likelihood of HUA among PLWH. The construction and application of the nomogram are illustrated in **Figure 3**. Each predictive factor was assigned a specific score according to its regression coefficient, and the total score was obtained by summing the individual scores of the two predictors. In this model, a higher total score corresponds to a higher risk of developing HUA following ART. To use the nomogram, a vertical line was drawn from each predictor’s value to the corresponding point axis to determine its score; the scores were then summed, and a final line was drawn from the total score axis to the outcome axis to determine the predicted probability of HUA.

**Figure 4:**
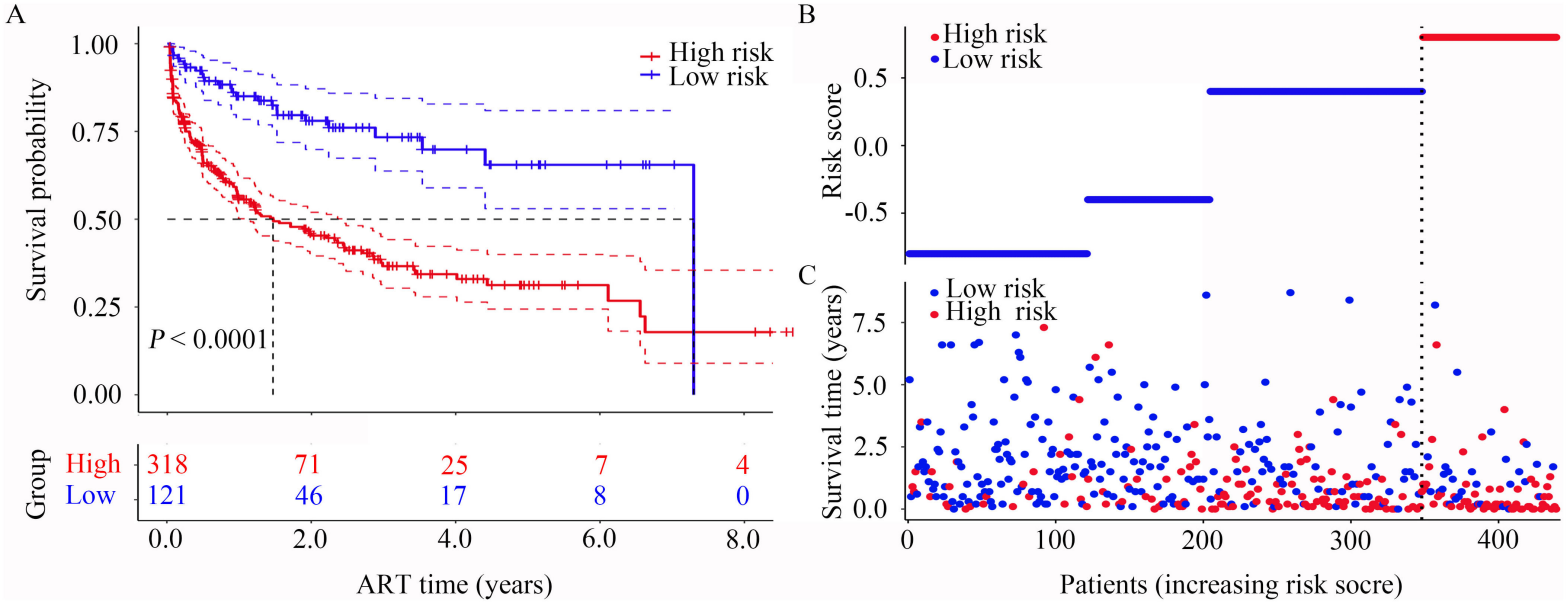
Construction of the risk score model with respect to PLWH with HUA. **(A)** Kaplan-Meier curves of the overall survival of the two HUA risk groups of PLWH with HUA. **(B, C)** Risk score curves and scatter plots of the risk of HUA among the PLWH cohorts. ART, antiretroviral therapy.

In our risk model, PLWH were divided into two groups (high and low risk), with the median of the calculated risk score serving as the criterion for division. Subsequent survival analysis revealed that the outcome of HUA differed significantly between the two groups (**Figure 4A**, *P* < 0.0001). HUA-free survival probabilities decreased as risk scores increased, which indicated that those in the extremely high-risk group faced the highest risk of HUA. Furthermore, the risk curve shows the relationship between HUA and the risk of PLWH. **Figures 4B, C** shows the risk values of PLWH in the two groups.

**Figure 5:**
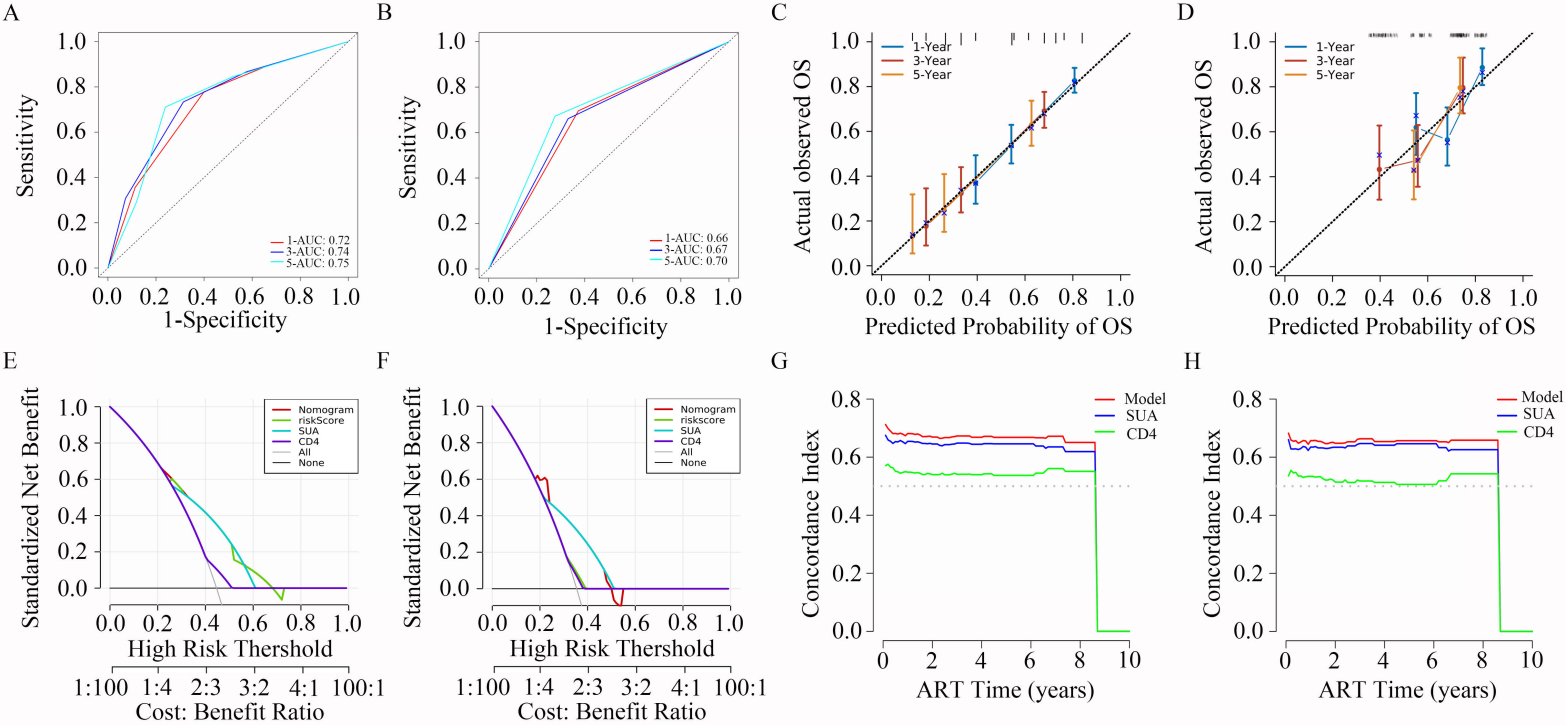
Calibration plots, DCA curves, C-index for the predictive nomogram. **(A, B)** Time-independent ROC curves of the nomogram for 1-, 3- and 5-year overall survival prediction in the training **(A)** and validation cohort **(B)**. **(C, D)** Calibration curves for 1-, 3- and 5-year survival depict the calibration of nomogram in terms of the agreement between the predicted probabilities and observed outcomes of the training **(C)** and validation cohort **(D)**. **(E, F)** The results of DCA analysis for the nomogram in the training **(E)** and validation cohort **(F)**. **(G, H)** Time-dependent C-index of the nomogram model in the training **(G)** and validation cohort **(H)**. AUC, area under the ROC curve; OS, overall survival; SUA, serum uric acid; CD4, CD4+ T cell count; ART, antiretroviral therapy.

Time-independent ROC curve analysis was used to evaluate the discriminatory accuracy of the prediction model (26). The AUC values for predicting 1-, 3-, and 5-year HUA risk were 0.72, 0.74, and 0.75 in the training set, and 0.66, 0.67, and 0.70 in the internal validation sample, respectively (**Figures 5A, B**). Calibration plots were constructed to assess the concordance between predicted and observed outcomes (27). As shown in **Figure 5C, D**, the calibration curves at each time point (1, 3, and 5 years) exhibited a high conformance in predicting the occurrence of HUA in the PLWH cohort.

DCA is widely used for evaluating the efficacy of specific clinical approaches (28). Here, DCA revealed that compared with the clinical model, the risk score achieved a greater net benefit across a wide range (17% to 70%) of risk threshold probabilities (**Figures 5E, F**). The C-index, which measures the agreement between predicted risk scores and observed survival times (29), yielded an optimism-corrected value of approximately 0.7 based on 1,000 bootstrap resamples in both cohorts (**Figures 5G, H**), reflecting the model’s satisfactory performance.

### 3.4 Prevalence of abnormal metabolic indicators in high-risk and low-risk groups after follow-up

Based on the calculated risk scores, all PLWH were divided into two groups: an HUA high-risk group, consisting of 128 patients; and an HUA low-risk group, consisting of 503 patients. At baseline, no significant differences were observed between the two groups in the prevalence of hypertriglyceridemia (HTG), high total cholesterol (HTC), low HDL-C, high LDL-C, dysglycemia, or eGFR abnormalities (**Table 5** left).

**Table 5:** Comparisons of the abnormal metabolic indexes between high-risk and low-risk groups.

Variable	Baseline			Follow-up		
	Low risk	High risk	*P* value	Low risk	High risk	*P* value
HTG						
No	400 (79.5)	99 (77.3)	0.588	361 (71.8)	95 (74.2)	0.580
Yes	103 (20.5)	29 (22.7)		142 (28.2)	33 (25.8)	
HTC						
No	447 (88.9)	121 (94.5)	0.056	402 (79.9)	117 (91.4)	0.002
Yes	56 (11.1)	7 (5.5)		101 (20.1)	11 (8.6)	
Low HDL-C						
No	228 (45.3)	49 (38.3)	0.151	272 (54.1)	67 (52.3)	0.726
Yes	275 (54.7)	79 (61.7)		231 (45.9)	61 (47.7)	
High LDL-C						
No	480 (95.4)	127 (99.2)	0.081	471 (93.6)	124 (96.9)	0.159
Yes	23 (4.6)	1 (0.8)		32 (6.4)	4 (3.1)	
Dysglycemia						
No	346 (68.8)	97 (75.8)	0.122	341 (67.8)	92 (71.9)	0.374
Yes	157 (31.2)	31 (24.2)		162 (32.2)	36 (28.1)	
Abnormal eGFR						
No	467 (92.8)	117 (91.4)	0.580	389 (77.3)	88 (68.8)	0.043
Yes	36 (7.2)	11 (8.6)		114 (22.7)	40 (31.3)	

HTG, hypertriglyceridemia; HTC, high total cholesterol; HDL-C, high-density lipoprotein cholesterol; LDL-C, low-density lipoprotein cholesterol; eGFR, estimated glomerular filtration rate.

Notably, after follow-up, the prevalence of HTC in the high-risk group was significantly lower than that in the low-risk group, whereas the prevalence of abnormal eGFR exhibited the opposite trend. Additionally, no significant differences in the prevalence of HTG, low HDL-C, high LDL-C, or dysglycemia were observed between the two groups (**Table 5** right).

### 3.5 The effects of different ART-initiated regimens on the SUA levels over time among PLWH without switching ART regimens

Among 243 PLWH without switching ART regimens, there were 71 PLWH in the B/F/TAF cohort, 114 PLWH in the EFV-containing cohort and 58 PLWH in the DTG-containing cohort (**Supplementary Table 3**). The demographic characteristics were largely similar among three groups (*P* > 0.05), including age, sex, BMI, marital status, transmission route, smoking status, drinking status, baseline CD4, and baseline SUA (**Supplementary Table 3**). The GLMM analysis showed a significant decrease in SUA values over time in PLWH receiving EFV-containing ART regimens (*P* < 0.001; **Table 6**), comparing with PLWH receiving the B/F/TAF regimens. However, there was no obvious difference in SUA values over time between DTG-containing ART and B/F/TAF cohort (*P* = 0.676; **Table 6**). Additionally, Cox regression analysis identified that an EFV-containing ART regimen at initiation was significantly associated with a lower risk of HUA (**Supplementary Table 4**).

**Table 6:** The effects of different ART regimens on the SUA levels over time among PLWH without switching ART regimens (n = 243).

Variables	Beta value (95%CI)	*P* value
Intercept	358.03 (348.16~367.90)	< 0.0001
Time		
T4 vs T0	-16.56 (-35.08~1.96)	0.080
T3 vs T0	-6.61 (-23.28~10.06)	0.437
T2 vs T0	-9.11 (-21.11~2.89)	0.137
T1 vs T0	-8.84 (-19.69~2.00)	0.110
Regimens		
DTG-containing vs B/F/TAF	-2.61 (-14.88~9.66)	0.676
EFV-containing vs B/F/TAF	-29.15(-39.83~-18.47)	< 0.0001

T0, at baseline; T1, one-year follow-up; T2, two-year follow-up; T3, three-year follow-up; T4, four-year follow-up; DTG, Dolutegravir; EFV, Efavirenz; B/F/TAF, Bictegravir/Emtricitabine-/Tenofovir Alafenamide; CI, confidence interval.

## 4 Discussion

HUA, characterized by elevated SUA levels, is associated with the occurrence of CVD, a leading cause of morbidity and mortality in PLWH (1, 2, 30). This association necessitates the development of a risk model for predicting HUA risk in PLWH after initiating ART. In this study, we divided 631 participants into training and validation cohorts. Using both LASSO regression and multivariate Cox regression, we systematically constructed HUA predictive models, which were rigorously evaluated for their sensitivity, specificity, and calibration to determine the best model for further development into a nomogram scoring system.

Previous studies have identified several risk factors for HUA in PLWH, including current alcohol use and an increased respiratory rate (10). Despite this assessment of individual risk factors, few robust predictive models for HUA have been reported among PLWH. Our data revealed that an elevated baseline SUA level and a lower baseline CD4^+^ T-cell count were independently associated with the development of HUA. This finding aligns with prior research demonstrating that a lower baseline CD4^+^ T-cell count correlates with higher SUA levels, a trend that is maintained throughout the follow-up period after ART initiation (13). Our results demonstrated that PLWH with baseline CD4^+^ T-cell counts ≤ 270 cells/μL had a significantly higher risk of developing HUA than those with counts > 270 cells/μL, supporting that a low baseline CD4^+^ T-cell count is an independent risk factor for HUA in this population. Notably, a meta-analysis reported that baseline SUA levels were independent predictors of future cardiovascular mortality (31). Furthermore, we found that a baseline SUA level > 349.1 μmol/L was the strongest predictor in the HUA risk model. This indicated that an elevated baseline SUA level can serve both as a key indicator for predicting subsequent HUA in PLWH receiving ART and as an independent risk factor for HUA.

While several studies have developed prediction models for HUA in various populations (32–36), none have focused on PLWH. Our study addresses this gap by reporting the construction of a predictive HUA model for PLWH after ART, based on the two identified predictors: baseline CD4^+^ T-cell count and baseline SUA levels. Moreover, we demonstrate, for the first time, that these two parameters are independent predictors of HUA in this cohort. The nomogram built from this model was evaluated using the C-index and DCA, which confirmed its effectiveness in predicting HUA occurrence in PLWH. Crucially, because all predictors in our nomogram are routinely detected in clinical practice, their measurement is both easy and cost-effective, thereby making the dynamic assessment and subsequent optimization of HUA therapies feasible for PLWH.

The nomogram combining the two identified risk factors not only demonstrated good clinical predictive performance but also effectively allowed for the categorization of patients into high- and low-risk groups. Furthermore, a significant difference was observed in the prevalence of high TC and abnormal eGFR between these two groups. This finding supports that PLWH who are classified as at high-risk for HUA are more likely to develop chronic kidney disease (CKD), consistent with previous studies linking HUA to CKD (eGFR < 60 mL/min per 1.73 m2) (37) and incident metabolic syndrome (38). Notably, previous studies supported that higher serum uric acid level (>237 µmol/L) have a protective effect on neurological outcomes after acute ischemic stroke (39, 40). Another study showed that HUA patients exposed to a higher level of uric acid (>=428 µmol/L) could significantly increase the incidence risk of stroke (41). These data implied that in the absence of hyperuricemia, elevated uric acid levels within a certain range are beneficial to patients. However, once the threshold for hyperuricemia is surpassed, further increases in uric acid levels become detrimental. The optimal cutoff value of uric acid for predicting favorable versus poor outcomes requires further investigation. Consequently, patients in this group should undergo regular uric acid testing and receive more individualized treatment.

We acknowledge certain inherent limitations in our study. Firstly, its retrospective nature means that some observed associations may represent consequences of HUA rather than contributing risk factors. Additionally, the baseline viral load has a missing rate of 12.7% and was therefore not included in further analyses. Finally, our model lacks external validation using independent data sources, underscoring the need for further research to confirm its robustness across diverse cohorts. Future studies with improved coverage, including multiple centers and more female participants, along with long-term follow-up, could yield more accurate and generalizable findings.

## 5 Conclusion

In conclusion, our study provides a potential model for assessing the risk of HUA in PLWH. This model demonstrates promising calibration and discriminatory ability and possesses clear clinical utility. Future studies need to assess the association of HUA with renal function among PLWH to determine whether HUA is a potential modifiable risk factor for renal function decline in PLWH.

## Data Availability

The original contributions presented in the study are included in the article/**Supplementary Material**. Further inquiries can be directed to the corresponding authors.
