# Supplementary material for: Development and internal validation of a risk model for hyperuricemia among people living with HIV in Hangzhou, China: a retrospective longitudinal cohort study
Source: Front Endocrinol (Lausanne). 2026 Apr 16;17:1736775. doi: 10.3389/fendo.2026.1736775 (PMC13128676; doi:10.3389/fendo.2026.1736775)
Supplement: Supplementary file 1 [file Table1.doc]

**Table S1.** The cutoff values of predictors of outcome of HUA for PLWH.

| Characteristics | Cut-off  value | Sensitivity  (%) | Specificity (%) | *P* value |
| --- | --- | --- | --- | --- |
| Baseline SUA (μmol/L) | > 349.1 | 72.96 | 62.14 | < 0.0001 |
| Baseline CD4 (cells/μL) | ≤ 270 | 45.41 | 65.02 | 0.0378 |
| Height (cm) | > 166 | 86.73 | 22.22 | 0.254 |

Note: SUA, serum uric acid; CD4, CD4^+^ T cells; cm: centimetre.

**Table S2.** The univariate cox regression analysis for factors associated with the outcome of HUA for PLWH.

| Variables | HR (95% CI) | *P* value |
| --- | --- | --- |
| Education level |  |  |
| College/Associate degree vs  High school or below | 1.234 (0.838–1.818) | 0.287 |
| Bachelor’s degree or higher vs  High school or below | 1.297 (0.940–1.789) | 0.113 |
| Height (cm) |  |  |
| > 166 vs ≤ 166 | 1.589 (1.051–2.402) | 0.028 |
| Baseline CD4 (cells/μL) |  |  |
| ≤ 270 vs > 270 | 1.407 (1.062 - 1.865) | 0.017 |
| Dysglycemia |  |  |
| Yes vs No | 1.071 (0.788–1.455) | 0.662 |
| Abnormal eGFR |  |  |
| Yes vs No | 1.131 (0.688–1.861) | 0.628 |
| Baseline SUA (μmol/L) |  |  |
| > 349.1 vs ≤ 349.1 | 3.330 (2.425–4.574) | < 0.0001 |

Note: cm: centimetre; CD4: CD4^+^ T cells; eGFR: estimated glomerular filtration rate; SUA: serum uric acid; HR: hazard ratio; CI: confidence interval.

**Table S3.** Demographic characteristics of PLWH receiving different ART initiation (n = 243).

| Characteristics | B/F/TAF  (n = 71) | EFV-containing  (n = 114) | DTG-containing  (n = 58) | *P* value |
| --- | --- | --- | --- | --- |
| Age (ART initiation), years | 34.9±13.0 | 37.1±14.0 | 34.9±13.6 | 0.463 |
| Gender |  |  |  |  |
| Male | 66 (93.00) | 99 (86.84) | 52 (89.66) | 0.423 |
| Female | 5 (7.00) | 15 (13.16) | 6 (10.34) |  |
| BMI (ART initiation) | 21.8±2.2 | 21.8±2.7 | 22.4±3.5 | 0.342 |
| Marital status |  |  |  |  |
| Married | 12 (16.90) | 32 (28.07) | 13 (22.41) | 0.215 |
| Divorced or widowed | 7 (9.86) | 16 (14.04) | 4 (6.90) |  |
| Unmarried | 52 (73.24) | 66 (57.89) | 41 (70.69) |  |
| Transmission route |  |  |  |  |
| Heterosexual | 15 (21.13) | 35 (30.70) | 8 (13.79) | 0.074 |
| Homosexual | 43 (60.56) | 58 (50.88) | 42 (72.41) |  |
| Other | 13 (18.31) | 21 (18.42) | 8 (13.79) |  |
| Smoking status |  |  |  |  |
| Never | 38 (53.52) | 68 (59.65) | 37 (63.79) | 0.074 |
| Occasional | 8 (11.27) | 22 (19.30) | 9 (15.52) |  |
| Daily | 25 (35.21) | 24 (21.05) | 12 (20.69) |  |
| Drinking status |  |  |  |  |
| Never | 40 (56.34) | 68 (59.65) | 31 (53.45) | 0.903 |
| Occasional | 29 (40.85) | 44 (38.60) | 26 (44.83) |  |
| Daily | 2 (2.82) | 2 (1.75) | 1 (1.72) |  |
| Baseline CD4 (≤ 270cells/μL) |  |  |  |  |
| Yes | 31 (43.66) | 37 (32.46) | 24 (41.38) | 0.255 |
| No | 40 (56.34) | 77 (67.54) | 34 (58.62) |  |
| Baseline SUA (> 349.1μmol/L) |  |  |  |  |
| Yes | 30 (42.25) | 58 (50.88) | 31 (53.45) | 0.384 |
| No | 41 (57.75) | 56 (49.12) | 27 (46.55) |  |

Note: BMI: Body mass index; CD4: CD4^+^ T cells; SUA: serum uric acid; DTG: Dolutegravir; EFV: Efavirenz; B/F/TAF: Bictegravir/Emtricitabine- /Tenofovir Alafenamide.

**Table S4.** The analysis of risk factors associated with the outcome of HUA for PLWH without switching ART regimens (n = 243).

| Variables | HR (95% CI) | *P* value |
| --- | --- | --- |
| Regimens |  |  |
| EFV-containing (vs B/F/TAF) | 0.32 (0.15~0.72) | 0.005 |
| DTG-containing (vs B/F/TAF) | 0.64 (0.30~1.37) | 0.248 |

Note: HR: hazard ratio; CI: confidence interval; DTG: Dolutegravir; EFV: Efavirenz; B/F/TAF: Bictegravir/Emtricitabine- /Tenofovir Alafenamide.
